# Use of WHONET-SaTScan system for simulated real-time detection of antimicrobial resistance clusters in a hospital in Italy, 2012 to 2014

**DOI:** 10.2807/1560-7917.ES.2017.22.11.30484

**Published:** 2017-03-16

**Authors:** Alessandra Natale, John Stelling, Marcello Meledandri, Louisa A Messenger, Fortunato D'Ancona

**Affiliations:** 1 London School of Hygiene and Tropical Medicine, London, United Kingdom; 2 Brigham and Women’s Hospital, WHO Collaborating Centre for Surveillance of Antimicrobial Resistance, Microbiology Laboratory, Boston, Massachusetts, United States; 3 San Filippo Neri Hospital, Roma 1 Health Authority, Rome, Italy; 4 Department of Disease Control, Faculty of Infectious Tropical Diseases, London School of Hygiene and Tropical Medicine, London, United Kingdom; 5 National Surveillance Centre of Epidemiology, Surveillance and Health Promotion (CNESPS), Department of Epidemiology and Infectious Diseases, Istituto Superiore di Sanità, Rome, Italy; 6 Ministry of Health, General Directorate of Health Prevention, Rome, Italy

**Keywords:** antimicrobial resistance clusters, automated surveillance, infection control, outbreaks, WHONET SaTScan, Italian national surveillance

## Abstract

Resistant pathogens infections cause in healthcare settings, higher patient mortality, longer hospitalisation times and higher costs for treatments. Strengthening and coordinating local, national and international surveillance systems is the cornerstone for the control of antimicrobial resistance (AMR). In this study, the WHONET-SaTScan software was applied in a hospital in Italy to identify potential outbreaks of AMR. Data from San Filippo Neri Hospital in Rome between 2012 and 2014 were extracted from the national surveillance system for antimicrobial resistance (AR-ISS) and analysed using the simulated prospective analysis for real-time cluster detection included in the WHONET-SaTScan software. Results were compared with the hospital infection prevention and control system. The WHONET-SaTScan identified 71 statistically significant clusters, some involving pathogens carrying multiple resistance phenotypes. Of these 71, three were also detected by the hospital system, while a further 15, detected by WHONET-SaTScan only, were considered of relevant importance and worth further investigation by the hospital infection control team. In this study, the WHONET-SaTScan system was applied for the first time to the surveillance of AMR in Italy as a tool to strengthen this surveillance to allow more timely intervention strategies both at local and national level, using data regularly collected by the Italian national surveillance system.

## Introduction

Antimicrobial resistance (AMR) is considered a public health threat as it is increasingly hampering effective treatment of bacterial and fungal diseases worldwide [1,2]. According to the Global Report on Surveillance of Antimicrobial Resistance, rates of resistance are increasing in all World Health Organization (WHO) Regions in pathogens causing infections in both healthcare and community settings [3]. A better strategy to enhance surveillance and strengthen collaborations at a global level is needed in order to coordinate efficient control strategies and to complete the current gaps in surveillance caused by lack of standard methodologies for data collection and failure of data sharing at local, national and international levels [4].

Despite multiple efforts for harmonisation and centralisation of clinical data, lack of data standardisation and poor data accessibility still constitute a worldwide problem. There is also a current need for a standardised interpretation of microbiology data as exemplified by the recent breakpoint harmonisation process promoted by the European Committee on Antimicrobial Susceptibility Testing (EUCAST) [5]. Clinical microbiology reports represent an important resource for the detection of ongoing dissemination of resistant (and even susceptible) pathogens. In spite of this, they are often underutilised not only at local hospital level, but also in national surveillance systems or across countries [6].

With the aim to centralise and coordinate European surveillance of AMR, the European Centre for Disease Prevention and Control (ECDC) coordinates the European Antimicrobial Resistance Surveillance Network (EARS-Net), a network of national surveillance systems [7]. This network collects routine clinical antimicrobial susceptibility data from 28 European Union (EU) and two European Economic Area (EEA) countries (Norway and Iceland) concerning invasive isolates (blood and cerebrospinal fluid, CSF) of eight organisms considered of public health concern [8]. This network has promoted the regular collection of clinical data in the participating countries and further highlighted the need of a standardised data format. To address such a need, and to facilitate data sharing, the WHO Collaborating Centre for Surveillance of Antimicrobial Resistance based at the Brigham and Women’s Hospital and Harvard Medical School, Boston (United States) developed a software to manage microbiology test results, the WHONET software [9], free to download, (www.whonet.org/software.html) that allows data entry into a standard format, or via BacLink utility software, a conversion tool [10]. Thanks to the software’s automated data entry and its ability to handle large datasets as well as to rapidly generate trends and patterns, WHONET has become the official component of many national surveillance programmes and is now used as a support tool in up to 120 WHO member states [11].

As a further application, WHONET has embedded the free SaTScan software (www.satscan.org) developed by Martin Kulldorff together with Information Management Services, Inc. and supported by various United States’ National Institutes of Health for the detection of spatial and temporal data clustering, using spatial, temporal or space-time scan statistics [12]. This algorithm is designed to evaluate random distribution or spatial and temporal clustering of diseases and to test their statistical significance, applied to surveillance of diseases and their geographic/spatial determinants or prospectively to timely detect outbreaks [13,14]. In combination, the WHONET-SaTScan system allows for timely detection of clusters of AMR pathogens in space and time facilitating outbreak investigations locally in a single hospital [15], in the community [16], or at national scale for real-time surveillance purposes [17]. The system also enables to study transmission of resistance between wards [18].

In this study, the WHONET-SaTScan software was applied for the first time within the Italian surveillance system. Since 2001, Italy has in place a national antibiotic resistance surveillance project coordinated by the Istituto Superiore di Sanità (AR-ISS), based on sentinel microbiological laboratories, integrating more than 50 hospitals throughout the country. Approximately 20 laboratories have been part of a sub-network called MICRONET and until the end of 2014, automatically submitted clinical data every night to a central server [19]. MICRONET included clinical data for all bacterial pathogens and all kind of samples. Furthermore, in Italy, the WHONET-BacLink software was already used at national level to aggregate and analyse data collected from all the laboratories belonging to the AR-ISS, making the Italian system ideal for the application of the WHONET-SaTScan system. In this work, data collected retrospectively between 2012 and 2014 from one hospital in Rome were analysed using a simulated prospective method to detect statistically significant clusters of pathogens of public health importance. The alerts generated by this method were then compared with the ones generated by the detection method currently in place in the hospital to assess the validity of the WHONET-SaTScan for a possible future implementation within the surveillance of AMR in a real-time and predictive manner.

## Methods

### Setting of the study

San Filippo Neri Hospital (SFNH) is a public hospital, with predominant surgical activity, located in the northern urban area of Rome with a capacity of 457 beds. Control and response to infections are responsibility of a hospital infections control team (Commissione Prevenzione e Controllo delle Infezioni Ospedaliere, CPCIO), composed of clinicians, microbiologists and virologists, infection preventionists, pharmacists and nurses. The CPCIO is coordinated by the hospital’s health manager, and collects microbiology data, detects epidemiological alerts and implements standardised control measures within the hospital. A procedure called ‘EpiD’ is activated when the definition of an outbreak is met (‘three or more samples of the same organism, isolated from three different patients within 5 days in the same operating unit’; ‘3 by 5’ rule) and containment measures are then set in place.

### Extraction of microbiology data and susceptibility test results 

Microbiology data were extracted from the MICRONET database, using date of test request as main parameter and setting restrictions to location (SFNH) and time (between January 2011 and the most recent data available at the time of the study, i.e. 30 May 2014). Data fields extracted included laboratory identity (ID), patient ID, sex, date of birth, age, pathogen type, ward, institution code, department, ward type, specimen number, specimen date, specimen type, specimen code, isolate number, admission date and susceptibility test results which were further described qualitatively as resistant (R), intermediate (I) and susceptible (S) based on minimum inhibitory concentration (MIC) test results and assigned as per EUCAST breakpoints [20]. Data were then converted to WHONET compatible format using the BacLink software. During the conversion the dataset was restricted to the first isolate per patient – including outpatients and inpatients admitted to the hospital any time before specimen collection (i.e. with no distinction between hospital-acquired or community infections) – over a 365 days period and all R and I results were combined as ‘non susceptible’ (NS) for purposes of resistance phenotype analysis.

Resistance profiles were adapted to this setting by choosing a panel of antibiotics for the main groups of pathogens, according to SFNH’s frequency of performed/reported antimicrobial tests per each group. The number of tests was obtained by performing a per cent resistant-intermediate-susceptible (%RIS) analysis on a sample of data from January to June 2013, assuming consistency of testing protocols across years. A 75% frequency was chosen as cut-off value.

### Statistical analysis

The SaTScan cluster detection tool integrated into the WHONET software was used to retrospectively identify clusters of antimicrobial resistant pathogens in SFNH. SaTScan can identify clusters of cases in terms of spatial only, temporal only, or combined spatial and temporal distributions. In this work, we used the SaTScan space-time permutation scan statistics for the evaluation of the statistical significance of identified clusters [14]. In this analysis, the temporal parameter was the ‘specimen date’ while the spatial parameters included a specific location within the hospital, such as the actual ‘ward’ or a group of wards with communal care characteristic defined as ‘service’. Non-spatial variables were the ‘pathogen type’ or ‘resistance profile’ based on antibiotic susceptibility test results. Clusters were identified using the categorical variables ‘pathogen type’, ‘resistance profile’, ‘ward’ and ‘service’ plus a combination of such variables. The statistical significance of clusters was evaluated by a Monte-Carlo maximum likelihood test using SatScan’s space-time permutation model. The parameters chosen for this analysis had been already assessed in previous studies [15,17]. A maximum cluster length of 60 days cut-off was chosen, corresponding to the maximum temporal scanning window size for signal generation. The statistical likelihood of signals is determined by the recurrence interval, which corresponds to the inverse of the p-value, expressed in days, signifying the time during which a similar signal would occur by random variation only. In this study, only clusters with a recurrence interval of > 365 days were included in the analysis. The baseline parameter (i.e. the temporal baseline preceding the maximum temporal window against which is compared) was set to 365 days. Thus data from 2011 were considered exclusively as baseline data (as they contributed to the first 365 days of the baseline) for the subsequent 2012 time period, and any clusters detected in 2011 were not included in the analysis.

### Dataset generation and comparison of WHONET-SaTScan results with the SFNH infection prevention and control system 

Overlapping signals generated by the WHONET-SaTScan analysis were combined into a single ‘signal cluster’. In particular, clusters including more information (more types of signal at the same time), more epidemiologically relevant (in terms of duration, number of cases etc.) and with higher recurrence interval, were chosen as representative clusters provided by the system. Cluster summary and cluster detail tables were generated and line listings of all the isolates involved in the alerts were also produced. The summary table of the alerts compiled by WHONET-SaTScan was compared with the CPCIO official list of microbiology alerts from 2012 to 2013 and an extract of the semester report of 2014. Because the CPCIO’s analysis of the alert reports from previous years revealed that more than 75% of all episodes within the hospital were caused by three pathogens: *Clostridium difficile*, multidrug resistant (MDR) *Acinetobacter baumannii* and carbapenem-resistant *Klebsiella pneumoniae*, the latest hospital reports, including the ones covered in our study, were restricted to such pathogens. Moreover, as *C. difficile* was not included in the SaTScan-WHONET list of organisms at the time of this study, our comparison could only be based on *A. baumannii* and *K. pneumoniae*. 

A questionnaire, adapted from a Brigham and Women’s Hospital’s, was used to assess whether there were any clusters detected by the WHONET-SaTScan of epidemiological or clinical importance. These alerts were further classified according to the level of concern caused (1 – no concern, disregard; 2 – low concern, await more cases; 3 – moderate concern, action; 4 – high concern, action) and for moderate and high concern, on the type of action (1 – notify other members of the CPCIO to increase awareness; 2 – assess background frequency of organism; 3 – start investigating by assessing medical records to find a common source; 4– activate containment measures). The questionnaire was completed by the head of the microbiology and virology laboratory who was a member of the CPCIO at the time of this study.

## Results

### Dataset

The microbiology dataset from SFNH collected from the beginning of 2011 to the end of May 2014 included a total of 11,777 samples, of which 7,994 from 2012 and 2014 were included in the final analysis, while 3,783 from 2011 were used as baseline data only. Specimen types were mainly urine (37.2%), pus (20.0%), and blood (11.2%). Table 1 depicts a summary of isolates’ characteristics between 2012 and 2014. Overall, isolates included 139 species, the most common being *Escherichia coli* (n = 2,092, 26.2%), *Staphylococcus aureus* (n = 742, 9.3%), *Enterococcus faecalis* (n = 656, 8.2%), *K. pneumoniae* (n = 554, 6.9%) and *Pseudomonas aeruginosa* (n = 506, 6.3%).

**Table 1:** Characteristics of isolates from San Filippo Neri Hospital extracted from MICRONET, Italy, January 2012–May 2014 (n = 7,994 isolates)

Isolates characteristics	Number of isolates	Percentage
Year		
2012	3,419	42.7
2013	3,327	41.7
2014^a^	1,248	15.6
Sex		
Female	4,340	54.3
Male	3,616	45.2
Missing information	38	0.5
Specimen type		
Urine	2,972	37.2
Pus	1,598	20.0
Blood	893	11.2
Tracheal aspirate	578	7.2
Vaginal swab	367	4.6
Cervical test	254	3.2
Sputum	238	3.0
Aspirate^b^	203	2.5
Nasal swab	168	2.1
Throat swab	161	2.0
Others	562	7.0
Organism group		
Gram-negative	4,483	56.0
Gram-positive	2,984	37.3
Mycoplasma	272	3.4
Anaerobe	183	2.3
Fungi	57	0.7
*Mycobacterium* (non tuberculosis)	15	0.2
Department of origin		
Outpatient	2,720	34.0
Medicine	1,970	24.6
Surgery	1,767	22.1
Intensive/intermediate care unit	1,108	13.8
Obstetric/gynaecology	160	2.0
Neonatology	121	1.5
Haematology/oncology	85	1.1
Emergency	49	0.6
Psychiatry	14	0.2

^a^ Data are from the first 5 months of 2014 only.

^b^ Aspirates other than tracheal aspirates.

### Signals created by WHONET-SaTScan

The WHONET-SaTScan analysis generated a total of 287 signals from 2012 to 2014 grouped into 90 ‘cluster summaries’, among which some, overlapping in the spatial components of service/ward and resistance phenotype, were further merged manually into 71 final clusters. Table 2 shows the summary characteristics of the final 71 clusters. Of these: 18 were caused by *E. coli* strains mostly fully susceptible to all antibiotics except for three, one of which being an extended-spectrum beta-lactamase (ESBL) strain; 13 by *E. faecalis* with different combinations of resistance phenotypes; seven by *K. pneumoniae*, one of which with resistance to four different classes of antibiotics and one in the intensive care unit (ICU) caused by a carbapenem-resistant strain; four by *P. aeruginosa*, one in ICU by a possible extensive drug-resistant (XDR) strain; three by *S. aureus*, one being a meticillin-resistant *S. aureus* (MRSA) strain; two by *A. baumannii,* one of which involving 13 cases of an XDR organism over two months; lastly, two by *Enterococcus faecium* including one by an MDR strain and the other including two cases of a vancomycin-resistant (VRE) strain in a neonatology ward.

**Table 2:** Summary characteristics of clusters generated by WHONET-SatScan in San Filippo Neri Hospital, Italy, 2012–2014 (n = 71 clusters)

Cluster characteristics	Number	Percentage^a^
Total number	71	100
Average number of clusters per month	4.5	NA
Year		
2012	17	24.0
2013	42	59.1
2014	12	16.9
Pathogen type		
*Escherichia coli*	18	25.4
*Enterococcus faecalis*	13	18.3
*Klebsiella pneumoniae*	7	9.9
*Pseudomonas aeruginosa*	5	7.0
*Staphylococcus aureus*	4	5.6
*Acinetobacter baumannii*	2	2.8
Other	22	31.0
Type of alerts		
Ward and resistance profile	24	33.8
Resistance profile	21	29.5
Service^b^ and resistance profile	16	22.5
Service^b^	4	5.7
Pathogen type	4	5.7
Ward	2	2.8
Mean number of signals per cluster (95% CI)	1.73	(1.53–1.93)
Number of cases		
Total	700	100
Median per cluster (range)	4	(2–143)
Cluster length in days		
1	10	14.1
2–5	17	24.0
6–10	10	14.1
11–50	21	29.5
> 50	13	18.3

CI: confidence interval; NA: not applicable.

^a^ Unless otherwise specified in the row heading.

^b^ A group of wards with communal care is defined as ‘service’.

### Comparison of alerts generated by WHONET-SaTScan with the hospital response system

In order to assess the validity of the method we compared the signal alerts generated by our analysis with the ‘EpiD’ procedure activated by the CPCIO. The total number of potential outbreaks detected by the WHONET-SaTScan system per year was higher than the number of activated ‘EpiD‘ (respectively, including *C. difficile* in ‘EpiD’, 17 vs 4 in 2012, 42 vs 6 in 2013 and 12 vs 4 in 2014). Table 3 summarises the comparison between the two systems, by year. In 2012, of two alerts detected by CPCIO (i.e. two activated ‘EpiD’), only the one involving *K. pneumoniae* is possibly in common between the two systems. However, this cluster was detected by WHONET-SaTScan in a different ward (outpatient) than by the CPCIO (which found the cluster in the ICU) and at a later time. As the CPCIO detected the *K. pneumoniae* cluster 11 days earlier, this outbreak was probably contained as result of the activation of the ‘Epid’ procedure. In 2013, three outbreaks were detected with a 100% agreement between the CPCIO and WHONET-SaTScan; one of these outbreaks involved *A. baumannii* in a cluster of long duration, which lasted from 20 May 2013 to 1 August 2013 with a recurrence interval of 2 years. This large outbreak, however, included a smaller signal outbreak clustered by service and resistance between 20 May 2013 and 25 June 2013 in the ICU with recurrence interval of 2.75 years (more rare) probably corresponding to the same signal that activated a response within the hospital. The signal of this cluster as generated by the WHONET-SaTScan is shown in the Figure. The other two outbreaks were caused by *K. pneumoniae* and seem to have activated the ‘EpiD’ procedure only months after the start of the outbreak, according to WHONET-SaTScan. In 2014, there was no official report from the hospital at the time of the study, and the alerts we could obtain were only from incomplete reports. However, none of the CPCIO alerts was detected by WHONET-SaTScan.

**Table 3:** Comparison between *Acinetobacter baumannii* and *Klebsiella pneumoniae* alerts detected by San Filippo Neri’s CPCIO and WHONET-SaTScan systems, Italy, 2012–2014

Year	Organism	Detected by the CPCIO				Detected by WHONET-SaTScan				Agreement between the two systems (%)
		Number of alerts	Date of activation	Ward	Number of cases^a^	Number of alerts	Start date	Ward	Number of cases	
2012	*Acinetobacter baumannii* MDR	1	14 Aug	ICU	≥ 3	0	NA	NA	NA	50
	*Klebsiella pneumoniae* MDR	1	11 Sep	ICU	≥ 3	1	22 Sep	OUT	2	
2013	*Acinetobacter baumannii* XDR	1	27 Jun	ICU	≥ 3	1	20 May	NSW	13	100
	*Klebsiella pneumoniae* MDR	1	11 Oct	ICU	≥ 3	1	6 May	ICU	6	
	*Klebsiella pneumoniae* KPC	1	27 Nov	ICU	≥ 3	1	3 Aug	ICU	3	
2014	*Klebsiella pneumoniae* KPC	1	Apr^b^	ICU	ND	0	NA	NA	NA	NA

CPCIO: Commissione Prevenzione e Controllo delle Infezioni Ospedaliere (hospital infections control team); ICU: intensive care unit; KPC: *Klebsiella pneumoniae* carbapenem-resistant; MDR: multidrug resistant; NA: not applicable; ND: no data; NSW: no specific ward; OUT:  outpatient ward; XDR: extensive drug-resistant.

^a^ The number of cases detected by the CPCIO is at least three to trigger the activation of control response as per outbreak definition (see text for details).

^b^ The exact date of activation was not available at the time of this study and only an unofficial report from 2014 was available.

**Figure:**
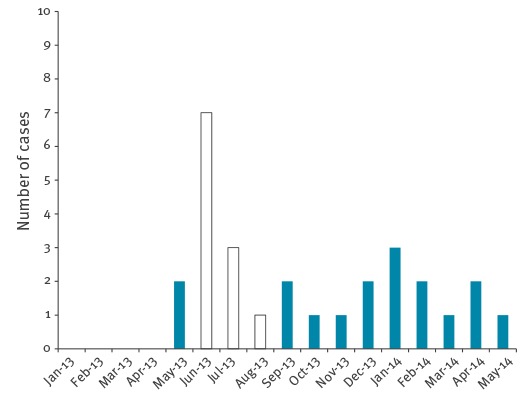
Distribution of *Acinetobacter baumannii* XDR cases and cluster alerts, San Filippo Neri Hospital, Italy, January 2013–May 2014

XDR: extensive drug-resistant.

The figure shows number of cases per month and the empty bars represent the signals that generated the alert in 2013 by WHONET-SaTScan; the sharp increase in number of cases in June 2013 compared with the previous months is statistically significant (see Table 4). The cases in July and August are part of the same cluster, as detected by the SaTScan temporal window and possibly part of the same epidemiological cluster.

### Evaluation of the alerts generated by WHONET-SaTScan

To assess the benefit of WHONET-SaTScan we asked a member of the CPCIO to evaluate the alerts generated by this system. Table 4 shows the alerts considered worth knowing by the hospital and by type of crucial characteristic (pathogen type, resistance phenotype or location). Of the 71 clusters detected by the WHONET-SaTScan analysis, 18 were considered of importance, of which only three had been initially detected by the CPCIO. The majority of these clusters were deemed relevant for the hospital because of the combined characteristics of pathogen type, resistance phenotype and location (n = 8, 44%), but also for their location alone (n = 4, 22%). Of these 18 clusters, including the ones detected by the CPCIO, six were considered of low concern, eight of moderate concern and four of high concern. For the eight alerts of moderate concern only two of the four types of possible actions were activated (i.e. 1 – notification of other members of CPCIO and 4 – start response measures), while the four alerts of high concern would trigger all four types of action. Among the high concern alerts, one caused by an *E. faecium* VRE strain in June 2013 and one by a *P. aeruginosa* MDR/XDR strain in July 2013, occurred completely undetected by the CPCIO.

**Table 4:** Cluster alerts detected by WHONET-SaTScan in San Filippo Neri Hospital considered relevant by the hospital’s infection control system (CPCIO) and critical characteristics of the alerts for the evaluation, Italy, 2012–2014 (n = 18 alerts)

Organism^a^	Type of alert	Alert characteristics^b^	Dates of outbreak (start–end)	Recurrence interval (1/n years)	Observed cases	Observed/ expected case ratio	Time span in days	CPCIO evaluation
2012								
*E. coli*	Ward/res	Gen med Fully susceptible	5 Sep–22 Sep	1/1.14	18	2.51	19	Pat/res
*P. aeruginosa*	Ward/res	Vascular surgery CTX, SXT	12 Oct–22 Oct	1/1.25	4	7.02	11	Pat/res
*E. aerogenes*	Ward/res	Vascular surgery Fully susceptible	22 Oct–25 Oct	1/19.85	2	6.06	4	Ward
*S. marcescens*	Res	SXT	13 Nov–15 Nov	1/22.40	2	5.56	3	Pat/res
2013								
*E. coli*	Ward/res	Neuro-rehab	20 Apr–20 Apr	1/1.52	2	153.85	1	Ward
*K. pneumoniae*	Res	CTX, CAZ, CIP, GEN, TZP, SXT	6 May–6 Jun	1/23.69	6	46.15	32	Pat/res
*E. faecium*	Serv/res	Neonatology AMP, ERY, GEN, IPM, LVX, MFX, VAN	10 Jun–12 Jun	1/1.30	2	21.05	3	Pat/res/serv
*S. aureus*	Res	LVX, OXA, PEN	26 Apr–22 Jun	1/1.44	10	4.13	58	Pat/res
*P. aeruginosa*	Ward/res	ICU CTX, CAZ, IPM, MEM, TZP, SXT	15 Jun–1 Jul	1/1.44	3	23.08	17	Pat/res/ward
*S. marcescens*	Serv/res	ICU AMK	19 May–15 Jul	1/2.11	3	4.76	58	Pat/res/serv
*A. baumannii*	Res	CTX, CAZ, CIP, GEN, IPM, MEM, SXT	20 May–1 Aug	1/2.00	13	3.56	74	Pat/res
*K. pneumoniae*	Serv/res	ICU AMK, CTX, CAZ, CIP, GEN, IPM, MEM, TZP, SXT	3 Aug–7 Aug	1/2.78	3	38.96	5	Pat/res/serv
*P. aeruginosa*	Serv	Interm care unit	26 Aug–27 Sep	1/4.13	4	9.52	33	Serv
*K. pneumoniae*	Serv	Interm care unit	5 Oct–18 Oct	1/1.25	3	15.79	14	Serv
*S. aureus*	Ward/res	Ortho-Trauma Fully susceptible	2 Dec–2 Dec	1/2.11	2	142.86	1	Pat/res/ward
*S. marcescens*	Serv/res	Gen Med AMK	24 Dec–3 Jan 2014	1/1.37	2	9.09	11	Pat/res/serv
2014								
*P. aeruginosa*	Serv/res	Surgery CIP, IPM, SXT	31 Mar–31 Mar	1/2.49	2	105.26	1	Pat/res/serv
*S. maltophila*	Ward/res	ICU	14 Apr–28 May	1/3.26	2	4.35	45	Pat/res/ward

Gen med: general medicine; ICU: intensive care unit; interm care unit: intermediate care unit; neuro-rehab: neuro-rehabilitation; ortho-trauma: orthopaedic trauma; pat: pathogen type; res: resistance; serv: service.

^a^ The organisms are abbreviated as follows: *A. baumannii: Acinetobacter baumannii; E. aerogenes: Enterobacter aerogenes; E. coli: Escherichia coli; E. faecium: Enterococcus faecium; K. pneumoniae: Klebsiella pneumoniae; P. aeruginosa: Pseudomonas aeruginosa; S. aureus: Staphylococcus aureus; S. maltophila: Stenotrophomonas maltophila; S. marcescens: Serratia marcescens*.

^b^ Antibiotics listed in this column are abbreviated as follows: AMK: amikacin; AMP: ampicillin; CAZ: ceftazidime; CIP: ciprofloxacin; CTX: cefotaxime; ERY: erythromycin; GEN: gentamicin; IPM: imipenem; LVX: levofloxacin; MEM: meropenem; MFX: moxifloxacin; OXA: oxacillin; PEN: penicillin; SXT: trimethoprim/sulfamethoxazole; TZP: tazobactam; VAN: vancomycin.

## Discussion

Timeliness is one of the main attributes of a good surveillance system, representing the ability to take appropriate public health action based on urgency [21]. Electronic data systems for the collection and analysis of microbiology data are becoming essential tools for surveillance to guarantee reliability, timeliness and standardisation across different compartments [22]. The aim of this work is to show the utility of a new tool, the WHONET-SaTScan, for surveillance of AMR in healthcare settings, especially in a context in which national surveillance programmes facilitate automated routine data collection, as the case of the Italian MICRONET [23].

When compared with traditional surveillance methods, the automated system used in this study showed a discrepancy in detected signals, as previously observed in other studies [15,24]. The higher number of signals produced by WHONET-SaTScan could be due to methodological differences compared to the CPCIO approach. WHONET-SaTScan generates a list of statistically significant signals, using an arbitrary choice for the cut-off value of significance (the recurrence interval), that affects sensitivity and specificity of the method, therefore meaning that statistically significant signals could not be necessarily indicative of a real outbreak or vice versa. Furthermore, the space-time permutation statistics cannot distinguish underlying fluctuations of local population sizes or temporal variations of detection frequency, leading to biased p-values [14]. In contrast, the CPCIO’s method is based on the classic definition of outbreak based on the ‘3 by 5’ rule, irrespective of the baseline incidence of the organism or the specific resistance phenotypes. In this case, its sensitivity is determined by the complexity of the case definition and personal interpretation, particularly in case of complex resistance phenotypes, while its specificity can be affected by baseline incidence. As a consequence, detection of clusters could be either delayed or even missing, especially if cases are spread throughout the hospital or, alternatively, infection control responses could be triggered when not needed, drawing staff and resources from the hospital and causing unnecessary distress to patients. On the other hand, traditional methods allow case-by-case interpretations based on personal experience and hospital background, identifying clusters not statistically but epidemiologically significant, like for example the cluster of *A. baumannii* in 2012, detected only by the CPCIO. Lack of information on the evolution of outbreaks after activation of the ‘EpiD’ procedure in the CPCIO reports, besides providing no indication on the efficacy of the measures adopted, interferes with the comparison between extent of outbreaks, as clusters detected by WHONET-SaTScan may result in higher case numbers and longer time spans.

The WHONET-SaTScan system showed some advantages compared to the CPCIO’s. The ‘3 by 5’ rule applied to a single ward at the time, in particular to critical care units, seems to be restrictive when compared with the WHONET-SaTScan ability to include groups of wards together or cover the whole hospital simultaneously. In this study, the ‘EpiD’ activated by the CPCIO occurred mainly in the ICU, while the clusters detected by WHONET-SaTScan were more homogeneously distributed throughout the hospital. The evaluation by the CPCIO coordinator showed that the main factor to trigger a response was the organism resistance profile, followed by pathogen type, location and source of specimen. The WHONET-SaTScan analysis allows for the investigation of clusters according to a specific resistance profile in combination to a specific location (‘resistance/ward’ and ‘resistance/service’), useful when an outbreak is occurring in a critical care ward. In addition, within the same analysis WHONET-SaTScan identifies clusters of susceptible strains, otherwise neglected due to a higher focus on resistance. Such clusters could be, in fact, of great interest to the infection control team for their routes of transmission and to the medical team in terms of pathogen characteristics and for offering different therapeutic options.

This study is not exempt of limitations and bias. Its retrospective nature undermines the efficacy of the WHONET-SaTScan system in the ‘field’. If conducted in real-time, it would have detected two clusters of MDR *K. pneumoniae* on average 126 days (95% confidence interval (CI): 66–186; n = 2), i.e. four months earlier, than the standard hospital control system, plus additional ones (two outbreaks of *E. faecium* VRE strain and *P. aeruginosa* MDR/XDR) that had occurred unnoticed within the hospital. The possibility to investigate prospectively the list of statistically significant alerts in combination with the clinical and epidemiological expertise of the hospital control team would provide a better evaluation of its benefits. Moreover, the inclusion of *C. difficile*, at the time not included in the list of organisms in the WHONET-SatScan analysis, would have better met the needs of the facility under investigation.

Reporting bias occurs as a consequence of selective reporting and control within the hospital due to a combination of resource availability, therapeutic choices and background prevalence data. For example, because of the endemic distribution in Italy of MRSA or ESBLs and the lack of appropriate resources for a prompt and effective intervention, the hospital adopted the policy of not reporting alerts triggered by these organisms. Again, the choice of antibiotics routinely used would reflect the panel of antibiotics tested and included in the configuration of WHONET, thus generating a list of alerts biased by the hospital policy on testing and reporting microbiology data. Lack of representativeness is another limitation of this study, as SFNH has in place an official procedure for infection control and a regular collection of standardised microbiology data, which most likely does not reflect the situation of other hospitals in Italy, a country with high between-hospital and regional variation.

Nevertheless, this work represents the first application of the WHONET-SaTScan system in a healthcare facility in Italy with the potential to be applied to other hospitals, extended to multiple hospitals in the same area or region or even on a larger scale to the whole national territory. Although the WHONET software is implemented within the surveillance systems of other European countries [25-27], this pilot study represents the first example of its application to the detection of clusters of resistant pathogens within a national surveillance system in Europe.
